# Risk Factors Associated with Injury and Mortality from Paediatric Low Speed Vehicle Incidents: A Systematic Review

**DOI:** 10.1155/2013/841360

**Published:** 2013-05-28

**Authors:** Anne Paul Anthikkat, Andrew Page, Ruth Barker

**Affiliations:** ^1^Discipline of Epidemiology and Biostatistics, School of Population Health, University of Queensland, Herston, QLD 4006, Australia; ^2^Paediatric Emergency Medicine Unit, Queensland Injury Surveillance Unit (QISU), Mater Health Services, Level 1 Whitty Building, Raymond Terrace, South Brisbane, QLD 4101, Australia

## Abstract

*Objective*. This study reviews modifiable risk factors associated with fatal and nonfatal injury from low-speed vehicle runover (LSVRO) incidents involving children aged 0–15 years. *Data Sources*. Electronic searches for child pedestrian and driveway injuries from the peer-reviewed literature and transport-related websites from 1955 to 2012. *Study Selection*. 41 studies met the study inclusion criteria. *Data Extraction*. A systematic narrative summary was conducted that included study design, methodology, risk factors, and other study variables. *Results*. The most commonly reported risk factors for LSVRO incidents included age under 5 years, male gender, and reversing vehicles. The majority of reported incidents involved residential driveways, but several studies identified other traffic and nontraffic locations. Low socioeconomic status and rental accommodation were also associated with LSVRO injury. Vehicles were most commonly driven by a family member, predominantly a parent. *Conclusion*. There are a number of modifiable vehicular, environmental, and behavioural factors associated with LSVRO injuries in young children that have been identified in the literature to date. Strategies relating to vehicle design (devices for increased rearward visibility and crash avoidance systems), housing design (physical separation of driveway and play areas), and behaviour (driver behaviour, supervision of young children) are discussed.

## 1. Introduction 

Low-speed vehicle runover (LSVRO) injuries have been variably categorized as “rollover,” “backover,” “driveway,” or other “nontraffic” pedestrian injuries. They predominantly occur in nontraffic (off-road) settings, but not exclusively so, and as such have different antecedents to high-speed vehicle and traffic-related pedestrian injuries. Investigation and reporting of off-road vehicular incidents vary by locality, and a lack of standardised definitions, coding, and reporting makes LSVRO incidents difficult to find in routine data sources.

Previous studies on child pedestrian injuries have identified specific contexts and antecedents relating to vehicular, environmental, and behavioural factors [1]. Injuries in children result in a significant economic burden to the health system due to long-term sequelae and ongoing disability amongst survivors [2]. In addition to the economic cost, there are significant psychological impacts on parents and carers [3, 4]. LSVRO incidents carry a significant risk of head and neck injury and have a high case fatality [5–8] with a l0-fold increase in mortality in children under 5 years of age [9].

Worldwide, studies on LSVRO incidents have been conducted over several decades [10–14]. These studies have identified a variety of sociodemographic, vehicular, environmental, and behavioural factors that predispose to LSVRO injury. However, there has been to date no peer-reviewed, systematic review on LSVRO incidents examining the consistency of these factors across different settings. The aim of this systematic review is to (i) identify common modifiable risk factors for paediatric LSVRO injuries across different settings and localities, (ii) identify specific risk factors that have been reported for particular settings or localities, and (iii) recommend potential countermeasures for reducing the frequency and severity of such events in the paediatric population.

## 2. Methods

### 2.1. Data Sources

Five electronic databases (PubMed, Scopus, CINAHL, Embase, and Web of Science) were searched for English language publications on child pedestrian injuries in the paediatric population for the period 1955-October 2012. Additional searches were also conducted using Google Scholar for unpublished studies, conference presentations, and reports, and also the databases of Transport Research Laboratory (TRL) in the United Kingdom (UK), Centers of Disease Control (CDC) in the United States (US), and National Highway Traffic Safety Administration (NHTSA) in the US. Secondary searches of reference lists were also conducted for potential articles.

### 2.2. Search Terms

The broad search term used was “child pedestrian injuries” and in subsequent additional searches included the keywords “driveway injuries” and “nontraffic injuries.” MesH terms used in Embase were (“child/exp” OR “child” OR “children/exp” OR “children” OR “paediatrics/exp” OR “paediatrics” OR “paediatrics/exp” OR “paediatrics” OR “infant/exp” OR “infant”) AND (“*driveway*” OR “*garage*” OR “*carport*”) AND (“accident/exp” OR “accident” OR “car/exp” OR “car” OR “automobile/exp” OR “automobile”).

### 2.3. Identification of the Literature

**Figure 1:**
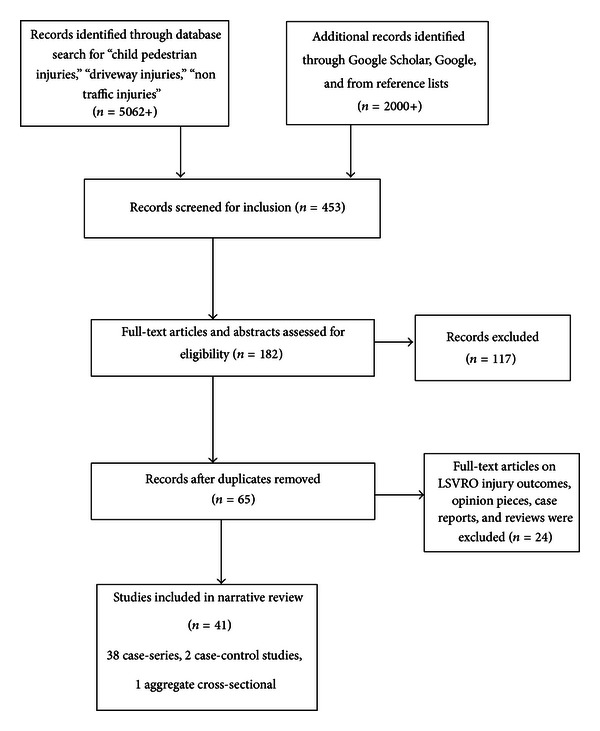
Steps for inclusion of articles in the systematic narrative review.

This review considered articles which mentioned injuries due to slow-moving vehicles, or vehicles reversing or rolling, or injuries occurring in various locations (e.g., driveways, parking lots, other nontraffic situations, and verge of traffic), or due to a child falling from a slow-moving vehicle. Titles and abstracts were read to identify papers for inclusion. Full-text articles, reviews, and reports were then obtained to extract relevant study factors (Figure 1). Assessment of eligibility of studies and extraction of data from study reports were conducted by authors APA and AP. Consensus was used to classify articles based on type of study design. Abstracts without full text, case studies, opinion pieces, and articles with information only on injury outcomes and treatments rather than antecedent factors were excluded. 

For this review, LSVRO cases were defined as children and adolescents aged 18 years or younger who sustained fatal or nonfatal injuries resulting from an impact with a motorised road vehicle moving at low speed either in a “nontraffic” setting (e.g., driveways, yard, garage, car park, and access road) or in a location where vehicles are moving into or out of traffic flow (traffic verge). Information on LSVRO incidents in the age-specific subgroups (0–5, 6–15, and ≥16 years) was extracted separately from studies as injury mechanisms that occurred in older children, adolescents, and young adults were different from those in young children.

### 2.4. Data Extraction and Synthesis

Data on key variables such as study design, methodology, risk factors, and other relevant variables for LSVRO injuries and fatalities were extracted using a standardised data extraction form. Studies were broadly classified as descriptive or analytic and individual level or aggregate (ecologic). For analytic studies, where groups exposed or not exposed to a given risk factor were compared, relative risk estimates (either OR or RR) were extracted. In descriptive studies, where no comparators were reported (e.g., in case-series studies) the number of cases (and proportion of total cases) with a given risk factor or characteristic was extracted. A systematic narrative summary of risk factors identified from each of these studies is outlined in this review. Meta-analysis was not appropriate due to variability in methods and measures between the studies; however identified studies could be grouped in terms of common domains relating to sociodemographic, vehicular, environmental, and behavioural factors.

## 3. Results

### 3.1. Identified Studies

Forty one articles met the inclusion criteria for LSVRO injuries predominantly in nontraffic settings and form the basis of this review (Tables 1–3). Of the 41 studies identified, ten studies investigated nonfatal injury (Table 1), 15 studies investigated fatal injury (Table 2), and 16 studies investigated both nonfatal and fatal injury (Table 3). Studies were predominantly conducted in the USA (16 studies), Australia (15 studies), and New Zealand (NZ) (8 studies); one study was conducted in Canada andone study reported from Austria. The majority of studies (38 of 41 studies) were case-series designs without a comparator group. There were two case-control studies (NZ) and one aggregate cross-sectional study (US). The majority of studies (31 of 41) appeared in the peer-reviewed literature, although ten government or commissioned reports were also identified.Fourcase reports on injury outcomes and treatment of driveway injuries, twofrom UK [46, 47] and twofrom USA [48, 49], were excluded. 

### 3.2. Sociodemographic Risk Factors

#### 3.2.1. Age

Twenty four of the 41 studies reported on LSVRO injuries in children aged up to 15 years, seven studies examined LSVRO injuries in children, adolescents, and young adults aged up to 20 years [7, 9, 13, 18, 28, 32, 41], and tenstudies [5, 8, 14, 23–27, 29, 42] reported solely on children aged up to 5 years.

Of the 31 studies where children older than 5 years were included, a higher proportion of LSVRO incidents were found in children aged ≤5 years when compared to children aged ≥5 years (range 35%–73% of cases). This was particularly noted for 16 of the 25 studies examining driveway-related LSVRO incidents. Within the 31 studies that included children aged up to 15 years, 15 studies [5, 14, 15, 20, 21, 23, 24, 28–31, 34–36, 42] noted that children aged 0–2 years (range 55%–73% of cases) comprised the highest proportion of LSVRO injuries.

#### 3.2.2. Sex

31 studies amongst the 41 studies investigated sex differences, the majority of which (27 of 31 studies) reported a higher proportion of injuries among boys (range 52%–86%). Twostudies on driveway incidents [7, 26] reported no sex differences, and two studies from US and NZ [14, 44] reported a higher proportion of girls than boys in nontraffic incidents mainly in driveways.Two studies showed a higher proportion of male children involved in LSVRO injuries that occurred in parking lots [21, 41].

When analyzed by mechanism,one study showed that LSVRO incidents caused by falling from a moving vehicle [8] involved a higher proportion of male children.   Nadler et al. reported a higher proportion of female children injured when the vehicle was moved by a child shifting the vehicle out of gear compared to when an adult was the driver [42].

#### 3.2.3. Race and Ethnicity

Of the 41 studies identified in the review, 11 studies investigated race and/or ethnicity as a factor associated with low-speed vehicle incidents [11, 12, 14, 15, 23, 24, 27, 36, 37, 40, 43].

Studies from NZ found that a higher proportion of driveway-related LSVRO injuries were reported in Pacific (range 26–49%) and Maori (range 25–48%) children [15, 24, 36, 37, 40]. Onestudy from the US found a lower proportion of cases in Hispanic compared to non-Hispanic children [12] while a recent US study reported a higher proportion of cases involving Hispanic children [14]. A case-series from US reported proportionately more cases among white children when compared to black children [11].

#### 3.2.4. Socioeconomic Status (SES)

Seven of eight studies [18, 22, 24, 25, 27, 37, 40, 43] that investigated SES found that LSVRO incidents were more common in lower than in higher SES groups as identified by area-based measures [24, 27], individual-level income, [43], or whether living in a rental property or not [18, 22, 37, 40, 43]. The strongest evidence for low SES as a risk factor comes from the case-control study of Roberts et al. from Auckland (NZ) [43]. Assessment of SES was based on parental interview and allocation of SES status according to parental occupation. 

#### 3.2.5. Number of Children in Household

One study [43] reported a 3-fold increase in risk for driveway-related LSVRO incidents where there were three or more children under the age of 5 years in one household compared to when two or less children were in the household.

#### 3.2.6. Driver Characteristics

Nineteen studies of LSVRO reported whether or not an adult driver was in control of the vehicle at the time of the incident (range 34–88%) and whether the driver was known to the child (range 41%–89%). However, several studies reported incidents that involved driverless vehicles [5, 6, 28, 38] or vehicles being inadvertently set in motion by children (range 10–15%) [7, 9, 18, 42]. In 18 of 23 studies investigating driver identity, a family member (parent, grandparent, sibling, or relative) was the driver (range 36–67%) and 13 of these studies reported proportions of cases where the driver was either the mother or father of the injured child. Amongst these, six studies [5, 6, 24, 25, 30, 37] noted that fathers (range 20%–40%) were proportionately more frequently involved in an LSVRO incident when compared to mothers. Other drivers identified were neighbours, friends, commercial drivers, and visiting tradesmen.

### 3.3. Environmental Risk Factors 

#### 3.3.1. Location of Incident

Thirty seven articles identified LSVRO injuries primarily on the basis of the nontraffic setting of the incident. Four studies also mentioned LSVRO injuries that occurred in traffic locations [25, 28, 30, 34]. Of the 41 studies investigating child pedestrian incidents in various locations, 15 studies investigated child pedestrian injuries relating to incidents in a range of locations with a subgroup of nontraffic incidents. 26 studies specifically focussed on child pedestrian incidents occurring in nontraffic locations. The most common nontraffic location investigated across these 26 studies was residential/home driveways with nine studies solely investigating paediatric LSVRO driveway incidents.

Of the 26 studies on driveways, 17 studies investigated nontraffic child pedestrian injuries relating to residential driveways (including friend's, neighbour's, and other residential locations) with proportions ranging from 17% to 93% [7–10, 13, 15, 17, 18, 26, 30, 35–38, 40, 42, 43] and nine studies specifically reported the child's “home driveway” as a common location of injury (range 17%–71%) [6, 14, 19, 23, 24, 28, 29, 32, 34]. 

Of the 41 studies, 15 studies reported the location of child pedestrian incidents occurring in nontraffic settings other than on a residential driveway: garages or carports [6, 30, 39], parking lots or car parks [16, 19–22, 28, 34, 41, 44], a holiday home [6], a company yard [6], a caravan park or camping ground [30], sidewalks [20, 28, 41], paddock on farm [30], other commercial premises [5, 6, 19, 30], and other off-road locations [28, 31, 33] and a general description of incidents occurring in “newer suburbs.” [10].

#### 3.3.2. Driveway Characteristics

Of 26 studies that included residential driveway-related LSVRO cases,  four studies identified driveways shared with another residence [15, 37, 40, 43] and one study identified having more than one parking area per driveway [15] as being more commonly associated with a driveway-related LSVRO incident. 

The twopopulation-based case-control studies from NZ [15, 43] showed a 3-fold increase in risk of driveway-related LSVRO incidents for dwellings with shared driveways [43] or more than one parking area per driveway [15]. The same degree of risk was evident for residences where there was no barrier separating the driveway from children's play areas [43], or where pedestrian access to the property was not separate from the driveway [15]. Driveways that exited onto a local road (compared to a cul-de-sac), driveways of longer length (>12 meters), and driveways that run along the boundary of properties were all associated with a greater than 3-fold increased risk of an LSVRO incident [15]. 

#### 3.3.3. Vehicle Risk Factors

*(1) Type of Vehicle*. Twenty six out of 41 studies investigated the type of vehicle involved in LSVRO incidents. Vehicle classification was not consistent across the studies. Passenger cars were reported to be involved in 18% to 71% of LSVRO incidents by twenty one studies; amongst them eight studies [6, 18, 23, 25–27, 30, 34] specified involvement of sedans (range 19%–69%).

Eighteen studies used a classification of four-wheel drive vehicles (4WDs or SUVs) and identified involvement of these vehicles in LSVRO incidents ranging from 8% to 47%.

Eleven studies identified the proportion of vans involved in LSVRO incidents [5, 6, 8, 17, 24, 27, 28, 34, 36, 37, 40] and nine studies [5, 8, 17, 23, 28, 32, 34, 36, 40] reported on the proportion of trucks or light transport vehicles (e.g., utility or pick-up vans) involved in LSVRO events (17% to 45%). Five studies combined the proportion of LSVRO incidents due to different vehicle types as follows: SUV and trucks [42], 4WD trucks and jeeps [5], 4WD and light commercial vehicles [13, 18], and SUV, vans, and trucks [14]. 

*(2) Direction of Vehicle*. Twenty six out of 41 studies examined vehicle direction at the time of LSVRO incident. Reversing vehicles accounted for varying proportions of LSVRO incidents across the 26 studies, ranging from 19% to 86%. Fourteen studies [6, 7, 12, 13, 16, 18, 20, 21, 23, 24, 30, 32, 34, 44] found that reversing vehicles accounted for more than 50% of incidents (range 26%–87%).

### 3.4. Behavioural Factors

#### 3.4.1. Child Behavior

*Eleven *out of 41 studies described the activity of the child at the time of the LSVRO incident [8, 11, 13, 18, 19, 23, 24, 26, 30, 31, 37]. Most commonly the injured child was involved in a “play” activity in the driveway area or was a “pedestrian.” Specific child activities prior to the LSVRO incident include playing in driveways or parking lots, playing with other children, playing under or behind parked cars, or playing within a vehicle, setting it in motion, and injuring a second child (external to, or fallen from, the vehicle).

#### 3.4.2. Driver Awareness of Children

Four studies discussed driver awareness [6, 21, 23, 37] of children involved in LSVRO incidents. Three studies reported that the majority of drivers were unaware of the presence of the child prior to the incident (range 58%–61%) [6, 21, 37]. Hsiao et al. [37] reported that 20% of drivers involved in a driveway LSVRO incident actively checked where the child was and surveyed the driveway before moving the vehicle.

#### 3.4.3. Adult Supervision

Six studies reported adult supervision of the child during the LSVRO incident [18, 21, 23, 24, 30, 31]. Agran and colleagues [21] noted that there was no adult supervision in 55% of driveway LSVRO incidents and that 36% of these children were injured while they were with other children, and the remaining 19% were alone at the time of the incident. In a survey of caregivers of children involved in driveway incidents, 58% perceived a lack of parental supervision as a contributing factor for the injury of their children [18]. In a recent report from New South Wales, 58% of carers did not know how the child came to be in the path of vehicle. In 33% of cases the children were with family members at the time of the incident and in 29% of cases “bystanders” observed the incident and tried to intervene [23].

### 3.5. Temporal Characteristics

Of the 41 studies, 18 investigated temporal characteristics, either time of day or week. Eight studies reported that LSVRO incidents most commonly occurred during late afternoon and early evening (3 pm–8 pm) [5, 12, 13, 17, 24, 37, 40, 44]. Three studies [6, 22, 24] reported a higher frequency on weekends, compared to weekdays.

## 4. Discussion

This systematic review provides a current assessment of antecedent factors associated with paediatric LSVRO injury and mortality and includes unpublished sources (such as government and commissioned reports) as well as peer-reviewed literature. This review highlights considerable variability in the reporting of risk factors for LSVRO incidents in children. A review of this topic is further complicated by the lack of a standard definition of LSVRO, limited reporting of nontraffic events in many countries, and the variability of case inclusion in the identified studies. All but two studies included in the review were conducted in three countries; the USA, Australia, or NZ. It is likely that cultural, vehicular, environmental, and behavioural factors influence the pattern of LSVRO incidents in each country. Although there are some consistent risk factors identified across many of the studies, it is difficult to generalize study findings beyond the study populations and even more problematic to extrapolate findings to middle- or lower-income countries. For example, there were no studies of LSVRO identified for regions in Asia and Africa where vehicular, environmental, and behavioural factors are likely to differ from studies in the USA, Australia, or NZ.

The literature on LSVRO incidents in children is dominated by case-series studies (38 of 41 studies) describing a range of possible sociodemographic, vehicular, environmental, and behavioural risk factors, and there is a paucity of controlled epidemiological studies validating these risk factors. Only two population-based case-control studies from one city (Auckland) in NZ [15, 43] and one population-based aggregate cross-sectional study [17] were identified. LSVRO may be an outcome more suited to descriptive case-series analysis given that it is a rare injury event, not routinely identified in coded surveillance systems, and with seemingly straightforward mechanisms. However population-based studies with comparator groups more reliably inform policy and prevention approaches, as such studies enable estimates of relative and attributable risk and test whether distributions of risk factors (particularly sociodemographic factors, driveway and housing characteristics, and vehicle types) are similar or different in children not experiencing LSVRO. 

The most commonly identified location for LSVRO incidents across all studies has consistently been the domestic driveway. However, it is not clear from the published literature whether this risk is inherent to the domestic driveway or due to the fact that young children (who are most vulnerable to this injury) spend most of their time in the home environment. However, two case control studies from NZ have specifically examined driveway characteristics. Shepherd et al. [15] identified driveway length, location, traffic speed at exit as well as separation of play areas, and pedestrian access from driveways as risk factors for driveway-related LSVRO incidents. Whether these risk factors (in themselves somewhat determined by urban development, housing design, and topography) are unique to the city of Auckland, where both studies were conducted, requires further investigation. 

The most consistent LSVRO risk factors identified across all studies for this review were child's age <5 years, male gender of child, and vehicle reversing at the time of the incident. Vulnerability of younger children (<5 years) to LSVRO incidents is likely to be due to a combination of rapidly developing mobility [12], and lack of perception of danger at this age, as well as anthropometric factors (such as height and weight relative to the vehicle). The higher frequency of LSVRO incidents involving young boys compared to girls reported in some studies may be due to differences in gender-related play activity and parental/carer risk perception which may be more likely to place boys in driveways and other outside areas.

The majority of studies that examined vehicle direction reported a higher proportion of LSVRO incidents involving reversing vehicles. The risk associated with reversing vehicles may in part be due to poor rear visibility in many vehicle types and models. However, forward visibility is also restricted in many vehicles. The environmental design feature of driveways and parking spaces, where vehicles predominantly nose-in on arrival and reverse out when leaving, may also contribute to the proportion of incidents involving reversing vehicles. Young children are likely to follow family members to vehicular areas when someone is leaving the residence. Risk associated with departing versus arriving vehicles has not been examined in the literature to date.

The relative contribution of 4WD or heavier vehicles to LSVRO incidents is difficult to gauge from studies considered for this review, due to the variability in classification and reporting of vehicle type. It is likely that any risk associated with such vehicles is due to both visibility to the front and rear of the vehicle as well as height and weight of the vehicle relative to the child's body, with smaller children more likely to go under taller vehicles without the driver seeing the child or noticing an impact. Many vehicles regardless of size or type have significant limitations to their forward and rearward visibility. The driver's rearward view whilst reversing is further complicated by a driver's physical limitations to turning and aberrations and blind spots when viewing the vehicle path in mirrors. Many vehicles currently on the market have reversing aids such as ultrasound sensors with alarms and rear cameras. Driver behaviour, in the use and interpretation of information from these devices, remains an important component in the control of the vehicle [50]. This review did not identify any studies that examined driver behaviour while reversing or the use of reversing aids as a separate point for analysis.

In a small number of studies, low socioeconomic status was associated with a higher proportion of LSVRO injury in children. Low socio-economic status is interrelated with other risk factors such as living in rental premises, shared driveways, a higher number of children in a household, low parental supervision, low parental education, and ethnicity. The best evidence for risk due to low SES status comes from a case-control study from Auckland, NZ [43]. It is not clear from the current literature whether this risk can be extrapolated to other localities. 

In this review, the driver was most commonly identified as being someone known to the child. This is particularly so for residential driveway LSVRO incidents, where the incident usually involved a parent or other family member as driver. Again, this finding is most likely explained by the frequency with which certain driver groups access the residence, with parents overrepresented. It is not clear from current studies whether there are gender or age differences in driver behaviour that might further contribute to risk. Identification of higher LSVRO risk for drivers exposed to young families allows targeted interventions for safer vehicle selection and driver behaviour. The high proportion of family members involved as drivers in LSVRO incidents also highlights a need for further exploration of the psychological impact of these incidents on families.

### 4.1. Implications 

#### 4.1.1. Environmental Factors

Several modifiable environmental risk factors have been identified in this review and include residential factors (house design, driveway design) and road factors (road network design). The strongest evidence exists for driveway-related LSVRO risk associated with long, shared driveways with direct child access and multiple vehicular and pedestrian users [15]. Currently, this evidence is limited to studies that originate from one city in NZ [15, 43]. Several studies have called for physical separation of driveways from children's play areas [7, 9, 13, 37, 38, 40, 43], separate pedestrian access to footpaths [8, 15, 30], and circular driveways [8, 13]. The implementation of the above strategies requires intervention from policy regulators through design and planning stages to construction. This requires long-term government and industry commitment to implement and is only likely to affect risk in new or recently renovated residences. 

#### 4.1.2. Vehicle Factors

Vehicle factors (vehicle size, type, design, and visibility) have all been discussed as potential risk factors for LSVRO incidents. However, there have been no studies to date that effectively evaluate vehicular risk factors. Although “4WDs” may be overrepresented in some LSVRO case series, what is needed is an estimate of exposure of families with young children to different types of vehicles in order to establish a clear risk association. It is likely that 4WDs and SUVs are popular choices for family vehicles, particularly where there is more than one child.

 Rear visibility is an independently modifiable risk factor, through the use of reversing aids [7–9, 13, 16, 17, 20, 30]. It is likely that insurance and market influence will drive vehicle manufacturers to make rear visibility aids standard rather than optional features with new vehicles. In part, this has been driven by consumer awareness groups, with some presenting results of comparative tests for rearward and forward visibility of vehicles commonly available on the market [51, 52]. At present, it is possible to retrofit rear visibility aids to many models of vehicle. What is not clear is to what degree standardization of rear visibility aids will affect the pattern and incidence of paediatric LSVRO incidents as there is also a significant behavioural component to the use and interpretation of information from these aids [50]. As with other safety innovations (seat belts, crumple zones, and airbags) emerging knowledge relating to car visibility indexes is likely to be incorporated into future car design, but this is a slower process. A proposition is underway in the United States for a new safety regulation to improve rearward visibility in every new vehicle by year 2014 [53]. A recent study from USA provides some evidence suggesting that reversing cameras when used appropriately (drivers glance at the system at the appropriate time) can successfully mitigate the occurrence of backing crashes into static objects, particularly when paired with an appropriate audible warning system [54].

It is not currently clear which performance requirements for rear visibility aids (location of the viewing screen, location of the camera, number of viewing screens and cameras, and field of camera view) would optimize the driver's rear view throughout the entire reversing process, for clearly the aid can only be effective if the driver is looking in the screen and can see the child. Similarly, due to the limitations of driver reaction time, rear visibility aids may not prevent LSVRO incidents where a child steps into the immediate path of a reversing vehicle.

More recent technological developments involve automated pedestrian crash avoidance systems, with some operating to the front of the vehicle and some to the rear [55, 56]. Again, some of these systems currently have room for human error as they can be overridden if the driver believes the vehicle to be stopping due to interference (terrain, landscaping).

#### 4.1.3. Behaviour Modification and Education

Few studies to date have reported on adult behavioural factors associated with LSVRO incidents [18, 31, 37]. A recent Australian survey reported that 77% of caregivers of children indicated that the driveway was a safe place, with more than half of the respondents sometimes using the driveway as a play area for children [57].

Government and advocacy organizations have attempted to raise awareness of risk factors associated with LSVRO incidents through print, television, and Internet sites [58–60]. These awareness campaigns are aimed at changing driver behaviour around several key messages: visually checking around the vehicle before moving, not allowing children to play within the vehicle, keeping children restrained within the moving vehicle, and never leaving children unattended in a vehicle. Again, more recent technological developments may reduce the risk of a child inadvertently releasing the handbrake, with some vehicles utilizing electronic handbrakes that require a special override manoeuvre for release when the driver seat belt is not engaged. 

As with any campaign aimed at changing behaviour, it is challenging to attain broad audience attention, and even more challenging to alter behaviour. This review has demonstrated that the majority of drivers involved in LSVRO incidents are family members or caregivers of the child; therefore, a targeted intervention may be more cost effective. However, even where there is awareness of the risks, supervisor and driver behaviour is likely to be affected by extraneous and competing factors, such as time pressure, distractions, and fatigue.

It is unlikely that an intervention aimed at child behavioural change to prevent LSVRO injury will significantly reduce injury in the peak age group (toddlers). Most toddlers do not have the perceptual skills to implement strategies [61] and the majority of incidents occur in a domestic setting, where they are unlikely to perceive risk despite direct instruction. A similar phenomenon is observed with toddler home pool immersion [62].

## 5. Conclusion

This study has reviewed available peer-reviewed and grey literature on LSVRO injuries in children and compared sociodemographic, vehicular, environmental, and behavioural factors reported within those studies. Most of the studies reviewed were descriptive case-series studies. The two population-based case-control studies that have been conducted have been restricted to driveway-related LSVRO incidents in one city and shown that there are sociodemographic (SES status) and environmental (driveway design, road design, and pedestrian access) risk factors associated with LSVRO injury. Additional studies employing analytic study designs together with standardised reporting and surveillance will improve upon this evidence base. Having started to identify modifiable risk factors, studies now need to be directed at implementing and evaluating interventions.

## Figures and Tables

**Table 1:** Studies of non-traffic low-speed vehicle runovers (LSVRO) and driveway runovers (DR) where the study outcome is non-fatal injury.

Study	Study period	Country	Study design^a^	Type of incident^b^	Age of children	Total cases	Study factors^c^													Main findings
							A	B	C	D	E	F	G	H	I	J	K	L	M	
Shepherd et al. [15]	2002–2006	NZ	CC	DR	<7 years	269	•	•	•						•				•	5-fold increased risk associated with a driveway exiting onto busy road 3-fold increased risk associated with driveways running along property boundary 3-fold increased risk associated with presence of additional parking 2-fold increased risk associated with driveway length >12 m 57% cases were males, median age 1 year

Nhan et al. [16]	1994–2003	Canada	CS	DR	0–13 years	49	•	•											•	69% of cases were aged 1–4 years 74% of cases were male 91% of cases admitted to hospital

Pinkney et al. [17]	1998–2003	US	AC	DR	<10 years	175	•	•				•	•	•	•		•	•		Incidence of 7.1 per 100,000 for driveway backovers in those aged <10 years 4-fold increased risk of driveway backover associated with minivan 2.5-fold increase risk of driveway backover associated with trucks 55% male children, in 48% of cases a family member was driver

Holland et al. [18]	2002–2005	Australia	CS	DR	<16 years	36	•	•			•	•	•	•	•	•	•	•	•	58% males; mean age = 48 months, 69% child's own home 85% -no separation from play areas 85% 4WDs and LCV 85% vehicle was reversing 81% driver known to the child 58% of parents of injured child attributed lack of supervision leading to injury

CDC [19]	2001–2003	US	CS	LSVRO	1–14 years	168	•	•							•	•			•	48% were children aged 1–4 years 55% were male children, 86% were pedestrian children as pedestrian sustained injuries at a rate 6 times (3.78) higher when compared to children riding a bicycle or tricycle (0.62) 67% were non-traffic events (2.67) 80% of injuries occurred at home (47.6%) or in a public property (31.9%) 40% of injuries occurred in driveways or parking lots

Mayr et al. [20]	1993–2001	Austria	CS	LSVRO	0–14 years	32	•					•	•	•					•	75% 0–5 years, 43% between 0–2 yrs 70% ran over by reversing vehicles or rolling backwards 63% were male children 37% occurred in residential driveways 44% cases cars driven by adult family members

Silen et al. [7]	1990–1994	US	CS	DR	<16 years	24	•	•				•		•	•				•	69% of cases involved adult driver, 15% involved older child or sibling 35% aged <2 years 50% of cases male

Agran et al.[21]	1987–1989	US	CS	DR	<15 years	39	•	•					•	•	•		•		•	74% of cases were males 56% were aged <2 years 78% of cases involved a reversing vehicle

Roberts et al. [22]	1992-1993	NZ	CS	LSVRO	<15 years	30	•				•				•			•	•	83% of cases occurred in driveways Median age was 1.5 years (range 0–7 yrs), male: female ratio 1.5 : 1 79% of cases occurred single dwellings with private driveways

Bell et al. [11]	1971–1979	US	CS	LSVRO	0–13 years	14	•	•	•			•			•	•			•	Mean age = 3 years, range 5 months to 13 years 12 cases (86%) were males 5 cases (36%) involved reversing vehicle by adult driver 4 cases (29%) involved unattended children

^a^Study design: CS: case series; CC: case control, AC: aggregate cross-sectional.

^b^LSVRO: non-traffic low-speed vehicle runover; DR: non-traffic low-speed vehicle runover occurring in domestic driveway.

^c^“^•^”indicates study conducts subgroup analysis of this study factor.

A: age; B: sex; C: race/ethnicity; D: SES; E: housing type; F: driver characteristics; G: vehicle type; H: direction of vehicle; I: environmental characteristics; J: behavioural characteristics; K: child anthropometric characteristics; L: temporal factors, M: peer-reviewed.

**Table 2:** Studies of non-traffic low-speed vehicle runovers (LSVRO) and driveway runovers (DR) where the study outcome is mortality.

Study	Study period	Country	Study design^a^	Type of incident^b^	Age of children	Total cases	Study factors^c^													Main findings
							A	B	C	D	E	F	G	H	I	J	K	L	M	
NSW CDRT Annual Report [23]	2002–2011	Australia	CS	LSVRO	<5 years		•	•	•			•	•	•	•	•	•			63% were 1-2 years; 58% were males Indigenous children were overrepresented 54% of cases the driver was the child's parent 29% were 4WD/SUV, 21% Sedans 57% of cases the vehicle was reversing 67% occurred in driveway and in the residential premises 58% of cases carer was unaware how the child reached near vehicle

Stark et al. [14]	1995–2010	US	CS	LSVRO	<4 years	31	•	•	•			•	•	•					•	Mean age 1.33 ± 0.23 years 67% were female, 67% Hispanics 67% drivers were family members or 33% acquaintance; 50% were cars; 50% SUV, van, or trucks 19% (6 cases) reversing vehicle All cases occurred in own driveway

Baker and White (Child and Youth Mortality Review Committee) [24]	2002–2008	NZ	CS	LSVRO	28 days–5 years	27	•	•	•	•		•	•	•		•		•		56% of cases between 12–23 months of age 70% were boys (19 boys and 8 girls) 48% were Maoris (14.8 per 100,000), 26% Pacific children (20.6 per 100,000) More deaths (~9) occurred in area with higher levels of deprivation (NZ Deprivation Index decile) 70% occurred on or around driveway, 37% of cases involved 4WD/SUV, 30% involved van and truck 18% cars, 63% reversing vehicle 88% were known drivers; 33% were by a father 37% of cases occurred between 5–7 pm, 48% cases occurred on a Saturday or Sunday

Griffin et al. [25]	2004–2008	Australia	CS	LSVRO	<5 years	15	•	•		•		•	•	•	•			•	•	87% were boys (12 boys and 3 girls) 86% of cases aged <5 years (4.8 per 100,000) 87% of cases occurred on private property 47% of cases involved 4WD vehicles 60% of cases involved a reversing vehicle No SES differences, cases predominantly occurred in rural areas

Byard and Jensen [26]	2000–2006	Australia	CS	DR	1–3 years	6	•	•					•		•	•			•	Mean age 16.8 months, male : female ratio 1 : 1 83% were reversing vehicle 66% in home driveway 66% of cases involved sedans

CCYPCG [27]	2004-2005	Australia	CS	LSVRO	<5 years	14	•		•	•			•	•	•					78% of cases aged 1–4 years 79% of cases occurred at family member's home 36% of cases involved 4WD vehicles 50% of cases involved a reversing vehicle 50% of cases occurred in rural or regional location

NHTSA [28]	1998–2002	US	CS	LSVRO	≤19 years	297	•					•	•	•	•					68% were children aged 0–2 years 45% incidents occurred in driveways 28% were cars, 17% pick-up trucks, 13% vans 29% were parents

QISU [29]	1994–2000	Australia	CS	LSVRO	<5 years	84	•	•				•	•	•	•			•		40% occurred in driveways 50% were aged 1 year. M : F ratio 1.5 : 1 60% of vehicles were reversing 41% of vehicles were 4WDs 54% involved relative or friend

Neeman et al. [30]	1996–1998	Australia	CS	DR	≤7 years	36	•	•				•	•	•	•	•	•	•		60% aged between 13–24 months 64% male children 78% occurred at child's residence 64% residential driveway 19% were cars, 22% were 4WDs, 22% utility vehicles 87% involved reversing vehicles 86% involved relative or friend as driver

Williamson et al. [31]	1995–2000	Australia	CS	LSVRO	<6 years	49	•	•					•		•	•				Majority aged 1-2 years, predominantly male (67%) 18% of cases occurred in residential driveway 58% of cases were not supervised at the time of the incident Majority of driveway runovers involved 4WDs

Byard et al. [32]	1977–1996	Australia	CS	LSVRO	1–16 years	68	•	•				•		•	•				•	4 cases (6%) occurred in home driveway 3 reversed over by car and 1 by truck 1 case driver was a parent Mean age = 2.5 (1.08), male : female ratio 1 : 3

Robinson and Nolan [6]	1985–1995	Australia	CS	LSVRO	≤15 years	28	•	•				•	•	•	•			•	•	68% of cases were males, age between 0.1–9.7 yrs 54% of cases occurred in home driveways 89% of cases involved driver known to child 57% of cases involved vehicle reversing 61% of cases driver was unaware of child

Olson et al. [33]	1986–1990	US	CS	LSVRO	0–14 years	21	•								•				•	Majority of cases occurred in those aged <5 years 33% deaths in non-traffic locations (driveways and parking lots)

Brison et al. [5]	1979–1983	US	CS	LSVRO	<5 years	41	•					•	•	•	•			•	•	73% of cases aged 0–2 years, 61% male children 73% of cases occurred in driveways 56% of cases involved a reversing vehicle 39% were light trucks, 29% 4WD vehicle

Kravitz and Korach [10]	1963-1964	US	CS	DR	0–9 years	9	•						•	•	•					Age range 13 months to 9 years, majority below 4 years Occurring in newer suburbs All incidents in driveways All incidents involved reversing cars

^a^Study design: CS: case series; CC: case control, AC: aggregate cross-sectional.

^b^LSVRO: non-traffic low-speed vehicle runover; DR: non-traffic low-speed vehicle runover occurring in domestic driveway.

^c^“^•^”indicates study conducts subgroup analysis of this study factor.

A: age; B: sex; C: race/ethnicity; D: SES; E: housing type; F: driver characteristics; G: vehicle type; H: direction of vehicle; I: environmental characteristics; J: child Behavioural characteristics; K: child anthropometric characteristics; L: temporal factors, M: peer reviewed.

**Table 3:** Studies of non-traffic low-speed vehicle runovers (LSVRO) and driveway runovers (DR) where the study outcome is nonfatal injury and mortality.

Study	Study period	Country	Study design^a^	Type of incident^b^	Age of children	Total cases	Study factors^c^													Main findings
							A	B	C	D	E	F	G	H	I	J	K	L	M	
Rice et al. [34]	2005–2007	US	CS	LSVRO	≤14 years	94	•	•				•	•	•	•			•	•	74% of cases were ≤4 years, 10% were fatal injuries In a subsample of 21 cases, median age −28 months; 62% were 1 or 2 years old (13 cases) 81% of cases were male (17 cases) 62% cases were back-over injuries (13 cases) 71% cases involved SUV, pick-up truck, or van 76% were drivers known to the child, 52% were parents 57% (12) occurred in driveways mainly home driveways 43% incidents occurred between 11 a.m.–5 p.m.

QTR [35]	2005–2009	Australia	CS	DR	0–14 years	53	•	•												Majority of cases aged <1 year 60% were male children

Hunter et al. [36]	2006–2009	NZ	CS	DR	0–15 Years	12	•	•	•				•		•					67% of cases aged <1 year (8 cases) 58% of cases were males (7 cases) 58% cases were Maori group (7 cases), all cases were driveway runover incidents. 3 cases were hit by car, 2 cases by a truck and a van

Hsiao et al. [37]	2001–2005	NZ	CS	DR	<15 years	93	•	•	•		•	•	•		•	•		•	•	80% of cases occurred at home 64% of cases used driveway as usual play area 73% of cases aged 0–4 years 68% of cases were Maori or Pacific groups

Davey et al. [38]	1998–2000	Australia	CS	DR	<15 years	76	•	•				•	•	•	•				•	40% of cases occurred in driveways 57% of cases were males 51% of cases aged <4 years 61% of cases involved cars 28% of cases involved a parent driving

Fenton et al. [8]	1997–2004	US	CS	DR	≤5 years	495	•	•					•	•	•	•		•	•	Younger children (≤5 yrs) accounted for 85% driveway injuries Median age −2.9 yrs 55% were cars Trucks (25%) carried highest mortality of 19% 66% between May and October 52% occurring Thursday and Saturday 56% between 3 p.m.–8 p.m.

QISU [39]	1998–2003	Australia	CS	LSVRO	<15 years	539	•								•					Driveways were common location (17%) for lowspeed run over in children aged <5 yrs 26% of cases a reversing vehicle was involved

Murphy et al. [40]	1998–2001	NZ	CS	DR	<15 years	76	•	•	•		•	•	•	•	•			•	•	86% <4 years old; 58% boys 74% were Maori and Pacific Islander groups 71% were cars, 20% van and light trucks 71% home driveway, 21% in shared driveways 68% reversed over by relative 84% of events occurred in rental property

Di Scala et al. [41]	1995–2000	US	CS	DR	<20 years	421	•	•												68% of children in driveways were 0–4 years 60% occurred among male children

Nadler et al. [42]	1986–1999	US	CS	DR	Mean age 3.37 years	64	•	•				•	•	•			•	•	•	80% of cases involved reversing vehicle 48% of cases involved an SUV or truck; 41% involved cars 52% of cases were males, mean age = 3.37 ± 2.4 34% of cases <2 70% were vehicles driven by an adult driver

Holland et al. [13]	1988–2000	Australia	CS	DR	<16 years	55	•					•	•	•		•		•	•	76% of cases were male 76% of cases involved reversing vehicle 71% of cases involve parent/relative as driver 55% of cases involved cars

Partrick et al. [9]	1991–1996	US	CS	DR	<18 years	51	•	•				•					•		•	80% of cases <5 years, 12% were between 5–9 years 59% of cases were male 34% of cases involved parent as driver 37% child was playing under or behind parked vehicle 20% children walking behind moving vehicle

Roberts et al. [43]	1992–1994	NZ	CC	DR	<15 years	212	•	•	•	•	•	•	•		•				•	3-fold increased risk associated with absence of physical separation of driveway from play area (PAR = 50%) 3-fold increased risk associated with shared driveway, households with 3 children or more, and child with NZ ethnic origin 2-fold increased risk associated with children living in rental accommodation, if child resided in the premises for <3 months

Roberts et al. [44]	1986–1990	NZ	CS	LSVRO	<15 years	99		•				•		•	•			•	•	7 of 8 fatalities occurred by a reversing vehicle in a driveway 5 children out of 8 deaths were females 93% of cases occurred in driveways 84% of cases involved a reversing vehicle 61% of cases involved a friend/relative driver

Stevenson et al. [45]	1980–1989	Australia	CS	DR	0–14 years	51									•				•	51 cases (4%) of total pedestrian injuries occurred in home driveways

Winn et al. [12]	1987–1989	US	CS	LSVRO	<15 years	58	•		•									•	•	38% of cases were aged 0–2 years 63% of Hispanic children were aged 3–5 years 43% of white children were aged 0–2 years

^a^Study design: CS: case series; CC: case control, AC: aggregate cross-sectional

^b^LSVRO: non-traffic low-speed vehicle runover; DR: non-traffic low-speed vehicle runover occurring in domestic driveway

^c^“^•^”indicates study conducts sub-group analysis of this study factor

A: age; B: sex; C: race/ethnicity; D: SES; E: housing type; F: driver characteristics; G: vehicle type; H: direction of vehicle; I: environmental characteristics; J: behavioural characteristics; K: child anthropometric characteristics; L: temporal factors, M: peer-reviewed.
